# Post-operative fracture risk assessment following tumor curettage in the distal femur: a hybrid in vitro and in silico biomechanical approach

**DOI:** 10.1038/s41598-020-78188-3

**Published:** 2020-12-07

**Authors:** Azadeh Ghouchani, Gholamreza Rouhi, Mohammad Hosein Ebrahimzadeh

**Affiliations:** 1 Faculty of Biomedical Engineering, Amirkabir University of Technology, No. 350, Hafez Ave, Valiasr Square, 1591634311 Tehran, Iran; 2 Orthopaedic Research Center, Department of Orthopaedic Surgery, Mashhad University of Medical Sciences, Ghaem Hospital, Ahmad Abad Street, Mashhad, Iran

**Keywords:** Orthopaedics, Biomedical engineering, Mechanical engineering

## Abstract

The distal femur is the predominant site for benign bone tumours and a common site for fracture following tumour removal or cementation. However, the lack of conclusive assessment criterion for post-operative fracture risk and appropriate devices for cement augmentation are serious concerns. Hence, a validated biomechanical tool was developed to assess bone strength, depending on the size and location of artificially created tumorous defects in the distal femora. The mechanics of the bone–cement interface was investigated to determine the main causes of reconstruction failure. Based on quantitative-CT images, non-linear and heterogeneous finite element (FE) models of human cadaveric distal femora with simulated tumourous defects were created and validated using in vitro mechanical tests from 14 cadaveric samples. Statistical analyses demonstrated a strong linear relationship (R^2^ = 0.95, slope = 1.12) with no significant difference between bone strengths predicted by in silico analyses and in vitro tests (*P* = 0.174). FE analyses showed little reduction in bone strength until the defect was 35% or more of epiphyseal volume, and reduction in bone strength was less pronounced for laterally located defects than medial side defects. Moreover, the proximal end of the cortical window and the most interior wall of the bone–cement interface were the most vulnerable sites for reconstruction failure.

## Introduction

Bone tumours can be classified as primary or metastatic. Primary tumours are divided into benign and malignant. Benign tumors like chondroblastoma or simple bone cysts usually do not affect life expectancy, but some, such as a giant cell tumour (GCT), can be aggressive, locally very destructive, and have a risk of malignancy^1^. GCTs account for 4–5% of all bone tumours and like chondroblastomas are mostly located around the knee or in the epiphysis of the long bones^2^. GCTs usually appear as lytic lesions and weaken bone due to their high tendency to resorb a considerable amount of tissue^3–5^. Load-bearing bones, especially the distal femur, are among the most affected anatomical sites and highly prone to fracture^6^. Surgery with extensive curettage is the accepted procedure for these kinds of tumours^6^. Unfortunately, the rate of post-operative fracture can be as high as 25% after curettage^7^. When the remaining defect is large, the patient can be at high risk of fracture and cement infilling and augmentation with internal fixation devices is common^6^. Since no satisfactory quantitative approach exits to determine the critical defect size, the decision for cementation is largely based on the surgeons’ clinical experience^6,8^. Some conservative criteria proposed for pathologic fracture risk assessment in patients with bone tumours, such as Mirels’ rating system^9^ or Harington’s criteria^10^, suffer from low specificity^5,11,12^. To date, no criterion has been quantitatively proposed to identify patients who are at high risk of post-operative fracture^13^. Thus, identification of the defects that need to be filled with bone cement and the appropriate device for cement augmentation remains imprecise. Defining a fracture risk criterion would benefit patients prone to fracture by regulating prophylactic interventions during tumour removal surgery while avoiding unnecessary treatment and stabilization for those with low fracture risk.

Bone strength has been shown to be the key determinant of fracture risk^14–16^. Information on bone geometry and its three-dimensional distribution of density, as well as the size and site of the tumourous defect, is needed to assess the bone strength. Thus, finite element analysis (FEA) based on quantitative computed tomography (QCT) images can be a powerful tool for predicting fracture risk ^17^. This technique provides an accurate determination of material properties, based on a volumetric measurement of bone density, and considers effective mechanical factors on bone strength^17^. Previous studies using the QCT-based FEA approach have reported promising results in predicting the strength of proximal femur ^16^ and vertebrae ^15^.

In the past few years, biomechanical approaches using FEA or rigidity analyses have been employed to improve the accuracy in predicting fracture risk in patients with a bone tumour and to decide on the most appropriate reconstruction method following tumour curettage^5,11,12,17–19^. Li et al. utilized a patient-specific linear QCT-based FEA method to compare the stiffness and stress distribution in a distal femur with a bone defect (which was left empty) with the femora filled with bone cement and augmented with an internal fixation device following GCT curettage^18^. In 2017, a biomechanical fracture risk criterion for tibia defects, in line with defect size and based on stress distribution, was introduced using linear FEA on homogeneous models of bones^5^. In 2018, an ad hoc CT-based FEA on a retrospective cohort of 45 patients with metastatic bone disease in the femur showed that 39% of patients who were referred by Mirels’ criteria (commonly used clinical criteria for predicting pathologic fracture risk in patients with a bone tumour) and underwent prophylactic stabilization may not have needed surgery^11^. A recent study employed a non-linear QCT-based FEA to predict patient-specific pathologic fractures in the proximal femora and confirmed this method’s ability to predict both failure loads and fracture locations by validating FEA results with corresponding in vitro tests on cadaveric specimens^17^. Recently, numerical methods using linear finite element (FE) models and structural rigidity analyses were used to predict failure loads of human distal femora following curettage and cementation^19^. Although several studies have investigated the fracture risk of a bone affected by a tumour, most concentrated on improving the accuracy of predicting pathologic fracture, but not the post-operative fracture risk^5,11,12,17^. Moreover, most of these studies suffer from low accuracy due to a lack of consideration of non-linear effects^5,18–20^, or ignoring non-homogeneous bone material properties^5^, or the lack of validation of FEA results with experimental or clinical data^5,18^. Therefore, there remains a great need for an accurate and validated biomechanical study on post-operative fracture risk assessment, particularly for defects in the epiphyseal region of the distal femur, the most vulnerable site for primary bone tumours, such as GCTs^6^. Such a biomechanical approach should quantitatively determine the necessity and the method of defect reconstruction following tumour removal, which is a controversial issue among specialists^6^.

While some researchers have reported good results when leaving the defect empty^21,22^, to be filled with the natural tissue from the bone healing process, others recommended bone reconstruction with PMMA bone cement following tumour curettage, due to its advantages in providing bone stability and preventing tumour recurrence^23–26^. Nonetheless, a retrospective study has shown that the rate of fracture following curettage is highly related to the size and location of the defect^22^. Therefore, the decision on whether or not cementation should be applied depends on the location and the size of the defect. Moreover, when cement augmentation with an internal fixation device is advised to reduce the risk of post-operative fracture, the best device to employ also remains a controversial issue^6^, likely due to the lack of key information regarding the mechanisms of failure at the interface between the bone and cement. Most previous studies regarding the selection of the best devices for augmentation, whether solely in vitro^26–28^*,* or FEA^18^ investigations, were conducted without experimental validation or consideration of the effect of bone–cement interface mechanics. Hence, the obtained results are not easily trustable, and the issue remains debatable.

In order to have an accurate and reliable FE model of a bone that is employable in clinical applications, accurate material and structural properties must be assigned to the model, and the FE models should be validated by either experimental or clinical data. In this work, a non-linear QCT-based FEA was used to predict bone strength in the presence of a bone defect in the distal femur. The FE models were validated using the results of in vitro mechanical experiments on corresponding cadaveric specimens. The validated models were then used to find critical bone defects that required cementation to reduce the likelihood of post-operative fracture. Finally, the effects of the defect size and location on the fracture load were investigated. Due to the lack of information on the bone–cement interface, as well as its importance in determining the risk of fracture, a secondary goal of this work was to investigate the mechanics of the bone–cement interface (BCI) to find out its leading causes of failure. Identifying high risk and vulnerable regions at the BCI would help improve the choice and placement of internal fixation devices for cement augmentation.

## Methods

### In vitro tests on human cadavers: specimen preparation, QCT scans, and mechanical tests

**Figure 1:**
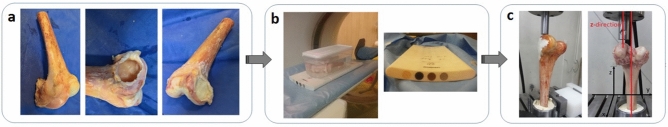
Various experimental phases of this study: (**a**) Specimen preparation, tumour surgery was mimicked in one sample of each pair while the contralateral femur remained intact; (**b**) QCT scan and the calibration phantom; and (**c**) Mechanical tests of a defective and an intact specimen. The compressive load was applied in the z-direction, parallel to the bone shaft.

According to the approval letter (#1397/768) from the Institutional Review Board (IRB) committee of Mashhad University of Medical Sciences, seven pairs of healthy human cadaveric distal femora (4 male and 3 female, mean age 37, ranging from 32–64 years old) were employed in this study. No musculoskeletal disorders, bone defects, or observable cracks were present in the femora used. A written consent form donating their body for research was obtained from voluntary donors during their lifetime, or from their next of kin after they died. All study procedures were conducted in accordance with permissions from the local ethics committee and the Declaration of Helsinki principles. A femur from each pair was randomly chosen to mimic tumour surgery by an orthopaedic surgeon, a defect eccentrically located in the epiphyseal part whose interior surface was smoothened using a high-speed burr, similar to what is usually performed in real tumour surgery^29–31^, was then created. The cavity was eventually filled with PMMA bone cement (Biomet Bone Cement R, Zimmer Biomet Co., USA). The contralateral femur was kept intact as the control sample (Fig. 1a). Each specimen was immersed in water to simulate soft tissue X-ray attenuation and to prevent beam hardening effects^32^, and was scanned using a clinical scanner (Siemens-Somatom 64, 140 kV, 80 mAs, 0.5 × 0.5 mm/pixel resolution, and 1 mm slice thickness), along with a calibration phantom (Mindways Soft-ware, Inc., San Francisco, CA), which was used to calibrate the resulting Hounsfield Units (HUs) to bone ash densities (${\rho }_{ash}$) (Fig. 1b).

After scanning, all specimens were positioned in a testing machine (Dynamic Testing Machine, Hct400/25, Zwick/Roell) with the bone diaphysis aligned in the z-direction, i.e., the direction perpendicular to the ground. A compression load with a displacement rate of 1 mm/min^33^ was applied via a 25 mm-diameter actuator on the medial condyle of the specimen parallel to the shaft axis while the proximal end of the specimen was embedded in bone cement to restrain the specimen in all directions (Fig. 1c). The actuator reaction force versus its displacement was drawn and the maximum force of the curve, as the fracture load of each specimen^16^, was recorded (F_Exp_). All steps including specimen preparation, QCT scans, and mechanical tests were carried out within 24 h, in order to decrease the risk of bone damage and changes in its material properties due to freezing and defrosting.

## Finite element modelling

### Geometry, mesh generation, and material assignment

**Figure 2:**
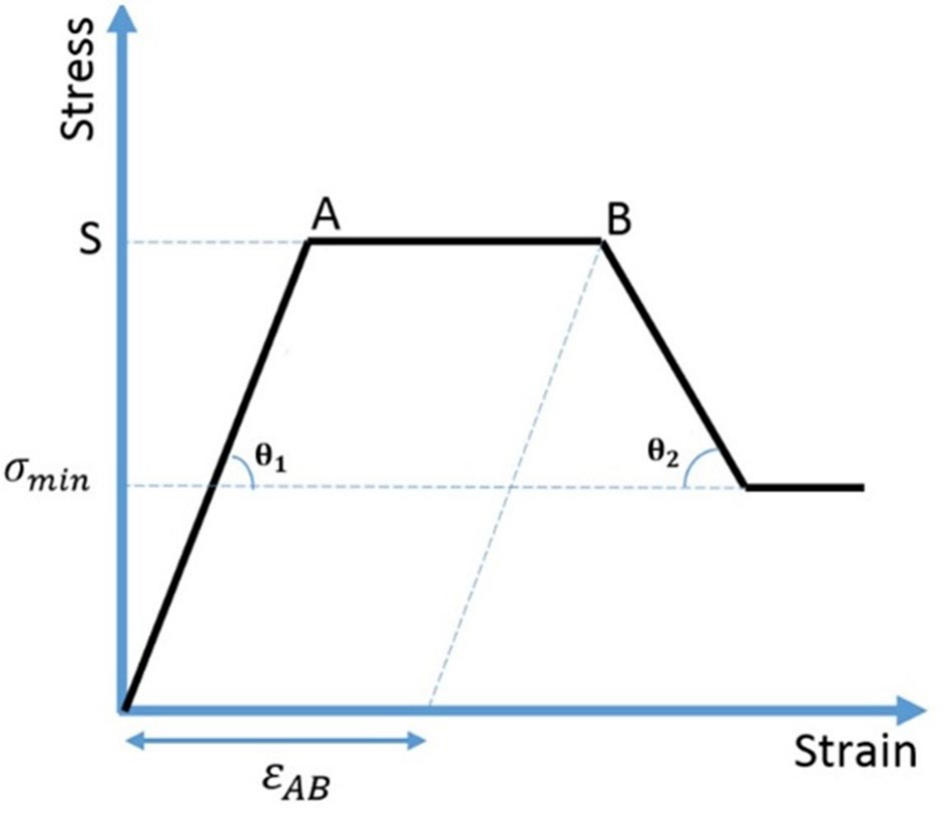
Quad-linear behaviour considered for bone: an elastic phase with an elastic modulus of $\mathrm{E}$; a perfect plastic phase with a plastic strain ${\upvarepsilon }_{\mathrm{AB}};$ a softening phase with a plastic modulus of $\mathrm{Ep};$ and a second perfect plastic phase.

Finite element models were generated using 3D modelling software (ScanIP and ScanFE, V. 3.1, Simpleware) by converting each voxel of the 2D images of QCT into an 8-node brick element. Heterogeneous material properties, based on density values, were assigned to the bone. A quad-linear behaviour was considered for each cubic bone element, as is shown in Fig. 2, which included: an elastic phase with an elastic modulus of $E$ until stress reaches yield stress $S$, then a perfect plastic phase with plastic strain ${\varepsilon }_{AB}$, followed by a softening phase with a plastic modulus of $Ep$ until reaching minimum stress ${\sigma }_{min}$, and finally a second perfect plastic phase^16^. Isotropic material properties were assigned to each cubic element based on its ash density value using the following relationships proposed by Keyak et al. for the distal femur^16^: $\mathrm{E }\left(\mathrm{MPa}\right)=14900{\uprho }_{\mathrm{ash}}^{1.86}$, $\mathrm{S }\left(\mathrm{MPa}\right)=102 {\uprho }_{\mathrm{ash}}^{1.80}$, ${\upvarepsilon }_{\mathrm{AB}}=0.00189+0.0241{\uprho }_{\mathrm{ash}}$ for the trabecular, and ${\upvarepsilon }_{\mathrm{AB}}=0.0184-0.0100{\uprho }_{\mathrm{ash}}$ for the cortical bone, ${\mathrm{E}}_{\mathrm{p}} \left(\mathrm{MPa}\right)=-2080{\uprho }_{\mathrm{ash}}^{1.45}$ for the trabecular, and ${\mathrm{E}}_{\mathrm{p}} \left(\mathrm{MPa}\right)={-1000}$ for the cortical bone, and ${\upsigma }_{\mathrm{min}} \left(\mathrm{MPa}\right)=43.1 {\uprho }_{\mathrm{ash}}^{1.81}$. Homogenous material properties were assigned to elements belonging to the cement region, i.e., E = 2 GPa^34^ and ν = 0.28^35^, with the strength of 40 MPa^36^.

### Bone–cement interface modelling, boundary and loading conditions

**Figure 3:**
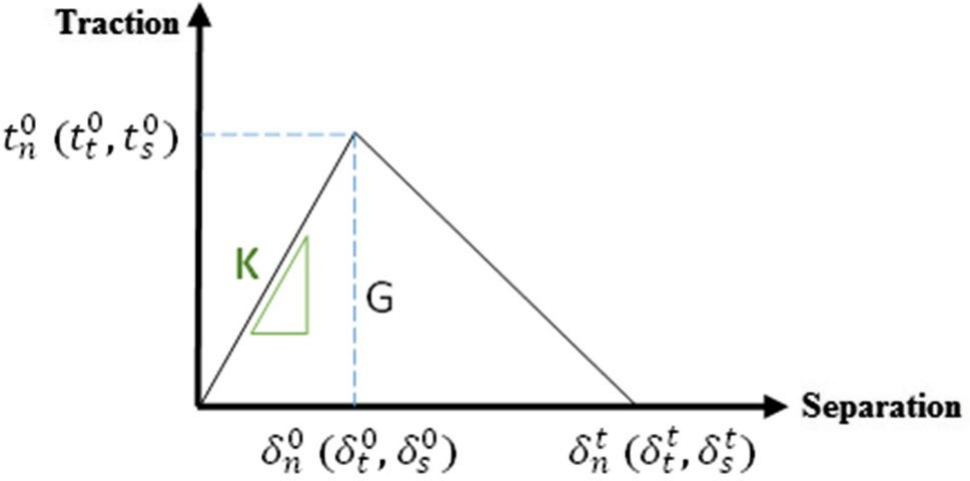
Traction–separation response considered for cohesive surfaces at the bone–cement interface (BCI), showing a linear elastic region with elastic stiffness (K) followed by initiation and evolution of the damage. The area under this curve is the fracture energy (G)^37^.

The cement was initially considered to be fully bonded to the surrounding bone, but could be separated when a damage criterion was met. To model this condition, two mesh-based surfaces were created on bone and cement at their interface. A surface to surface contact with small sliding and cohesive behaviour with damage modelling was defined. The damage was applied with a traction–separation model, which assumes an initial linear elastic behaviour, as shown in Fig. 3, followed by initiation and evolution of damage to the bone–cement interface property^33^.

In this model, the elastic behaviour was written in terms of an elastic constitutive matrix (K) that relates the normal and shear stresses (t) to the normal and shear separations (δ) across the interface (Eq. 1). Uncoupled traction–separation behaviour was considered, in which only the terms ${K}_{nn}, {K}_{ss}, and {K}_{tt}$ need to be defined^37^. Damage modelling was used to simulate the degradation and eventual failure of the bond between bone and cement, and its initiation was defined based on the maximum stress criterion that assumes onset of damage when the maximum contact stress ratio reaches the value of one, i.e., $f=1$ in Eq. (2). Damage evolution was defined with energy-based mixed-mode power-law fracture criterion (Eq. 3), which states that failure under mixed-mode conditions is governed by a power-law interaction of the energies required to cause failure in each mode (one normal and two shear stresses)^37^.

1 $$t=\left[\begin{array}{c}{t}_{n}\\ {t}_{s}\\ {t}_{t}\end{array}\right]=\left[\begin{array}{c}{K}_{nn}\\ {K}_{ns}\\ {K}_{nt}\end{array}\begin{array}{c}{K}_{ns}\\ {K}_{ss}\\ {K}_{st}\end{array}\begin{array}{c}{K}_{nt}\\ {K}_{st}\\ {K}_{tt}\end{array}\right]\left[\begin{array}{c}{\delta }_{n}\\ {\delta }_{s}\\ {\delta }_{t}\end{array}\right]=K\delta$$

2 $$f=MAX\left\{\frac{\langle {t}_{n}\rangle }{{t}_{n}^{0}},\frac{{t}_{t}}{{t}_{t}^{0}},\frac{{t}_{s}}{{t}_{s}^{0}}\right\}$$

3 $$\left\{\frac{{G}_{n}}{{G}_{n}^{C}}\right\}+\left\{\frac{{G}_{s}}{{G}_{s}^{C}}\right\}+\left\{\frac{{G}_{t}}{{G}_{t}^{C}}\right\}=1$$

In Eqs. (1–3), t represents the traction stress vector at the interface of the bone and cement, K is the stiffness matrix, $\delta$ represents the associated separation at the interface, G is the fracture energy (defined as the surface area under the traction–separation curve), ${t}^{0}$ is the peak value of contact stress, and ${\mathrm{G}}^{\mathrm{C}}$ refers to the critical fracture energy required to cause failure. The subscripts n, s, and t denote the normal and two shear directions, respectively. The values of stress at damage initiation ${\mathrm{t}}^{0}$, (MPa), and energy for damage evolution ${\mathrm{G}}^{\mathrm{C}}$ (N/mm) were taken from the experimental work of Mann et al.^38^ on deriving a failure model for the femur bone-PMMA cement interface as a function of the interdigitated bone, ${\mathrm{q}}_{\mathrm{int}} \left(\frac{\mathrm{mg}}{\mathrm{cc}}\mathrm{mm}\right),$ and load angle $\uptheta$ (in degrees):

4 $${\text{t}}^{0} = 0.811 + 0.002524.{\text{q}}_{{{\text{int}}}} + 0.0000268.{ }\left( {{\text{q}}_{{{\text{int}}}} .{\uptheta }} \right)$$

5 $${\text{G}}^{{\text{C}}} = 0.064 + 0.001578.{\text{q}}_{{{\text{int}}}} + 0.0000249.{ }\left( {{\text{q}}_{{{\text{int}}}} .{\uptheta }} \right){ }$$

Failure parameters for three different amounts of interdigitated bone ${\mathrm{q}}_{\mathrm{int}}$, 100, 263, and 381 mg/cc mm, based on previous FEA studies on the failure of the bone–cement interface^39–41^, were determined. By considering $\uptheta$ equals to 0° and 90° in Eqs. (4) and (5), respectively, the stress at damage initiation and energy for damage evolution for tension and shear were obtained. Elastic stiffness (MPa/mm) was derived by dividing stress at damage initiation to the corresponding displacements ${(\updelta }^{0}$), which are 0.07 mm in tension, and 0.082 mm in shear^39^.

**Figure 4:**
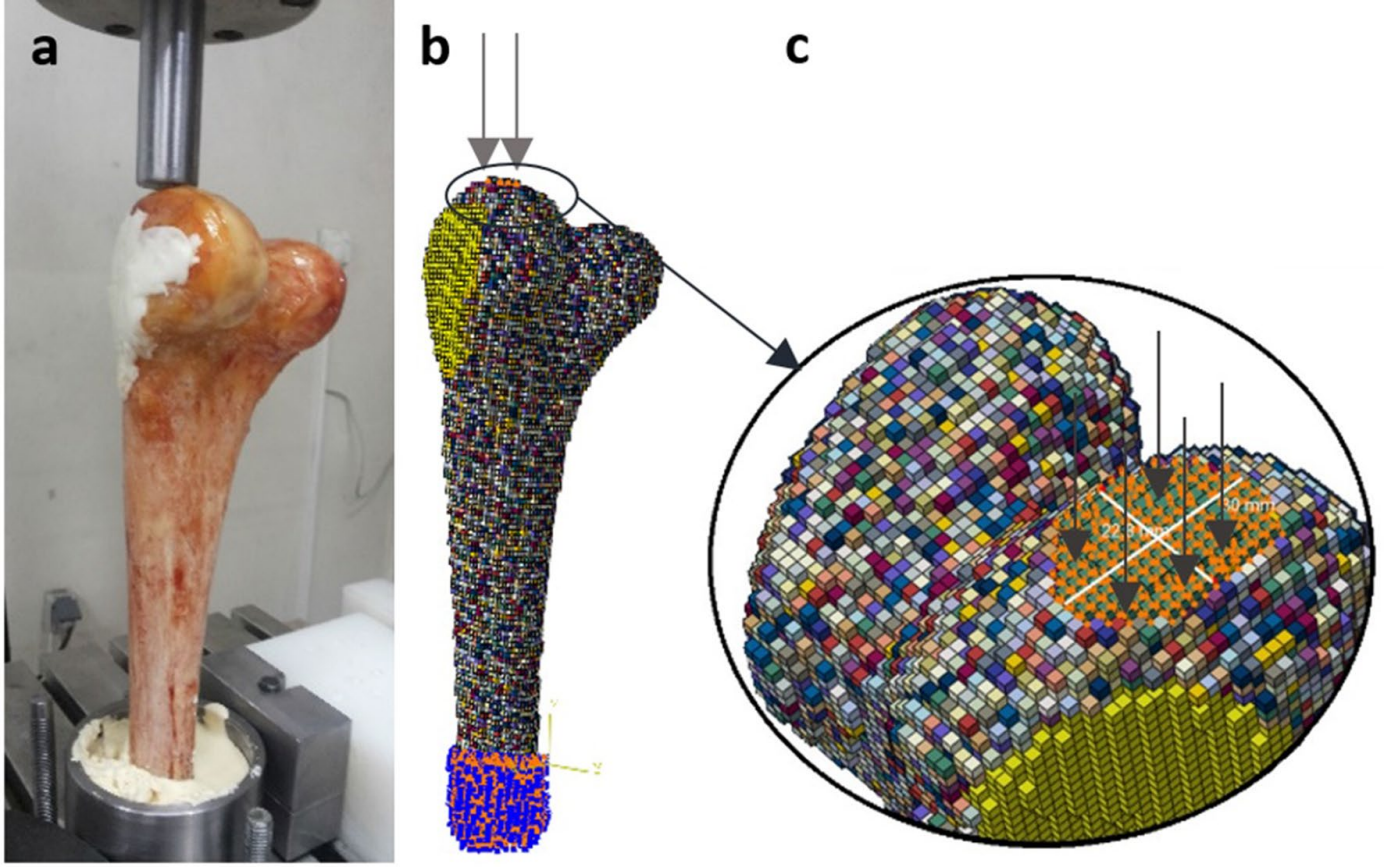
Boundary and loading conditions: (**a**) Set up for the in vitro tests, (**b**) FE models showing proximal end constrained and compression load applied on the medial condyle, and (**c**) The compression load was applied on nodes located on a circle with an approximate diameter of 25 mm.

In order to mimic the boundary conditions applied in the in vitro tests, the proximal end of the model was restricted in all directions, and a compressive load in the form of displacement was applied to the nodes located on a 25 mm circle on the medial condyle (Fig. 4).

To prevent element distortion during loading, an elastic modulus of 20 GPa and a yield stress of 200 MPa were assigned^14,16^ to the elements of the nodes where the displacements were applied (Fig. 4c). The sum of the reaction forces of the nodes (where the compressive load was applied) versus their average displacement was plotted, and the maximum force on the curve was considered as the FE predicted fracture load (F_FE_). Finite element analyses were performed in ABAQUS (v6.13-4, Dassault Systèmes), using distortion energy failure theory, as a validated bone failure theory^42,43^.

### Models validation and evaluation

Keyak et al.’ approach^16^ was used to evaluate and validate the FE models. The models were divided into two groups: 1-Tuning group (TG) with five femora, and 2-Evaluation group (EG) with the remaining nine femora. In the TG, the material properties of every element of all the models, namely, E, S, and E_p_, were reduced incrementally by 1%, using a home-made MATLAB code, and the bone strength was calculated by employing the procedure explained. Then, a paired t-test was applied to assess if the mean prediction error, $\mu ={F}_{FE}-{F}_{Exp}$, for the 5 pairs (FEMs-Experiments) of the TG was not different from zero. Reduction in the material properties was applied until there were no significant differences between bone strengths predicted by FEA and those found from in-vitro experiments.

In the TG, the material properties of all the elements of each model, namely, E, S, and E_p_ were reduced incrementally until the mean prediction error was not different from zero, based on a paired t test. The relations between material properties and bone density were derived for the superior-inferior (S-I) direction^16^. By assuming isotropic properties for bone, the FE models tend to overestimate the bone strength. Hence, the material properties which are dependent on the direction of the load were reduced to help account for the anisotropic behaviour of bone^16^. The final material properties employed on the TG that met the statistical test criterion were then assigned to the EG models. The precision of the FE models was evaluated by applying a paired t test for the EG models, as an independent data set. The relation between ${F}_{FE}$ and ${F}_{Exp}$ was computed using linear regression analysis, a paired t-test, and Pearson’s correlation coefficients. Statistical analyses were carried out using SPSS (V. 16, SPSS Inc., Chicago, USA). A P-value less than 0.05 was considered statistically significant.

### Defect construction in FE models

**Figure 5:**
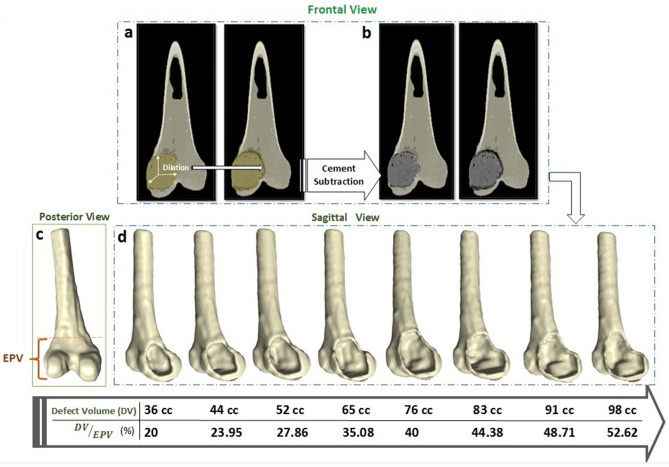
Defect construction in the FE models: (**a**) Dilation of cement region in all three directions; (**b**) Cement subtraction to create empty space; (**c**) Epiphyseal volume (EPV) calculated from the distal end to the beginning of the diaphysis, shown in the left distal femur; and (**d**) Creation of different defect volumes (DVs) ranging from 36–98 cc, or 20–52.62 percent of EPV.

Tumourous bone defects of different sizes, mimicking the defect following tumour curettage, were created in a validated FE model. A FE model constructed from a specimen with a defect volume of 36 cc in the medial condyle was used, and the defect area was dilated incrementally in all directions using the tools provided in Scan IP to create eight defect sizes, 36, 44, 52, 65, 74, 83, 91, and 98 cc (Fig. 5). The percentage of the condylar region occupied by the defect was also calculated by dividing the defect volume (DV) to the epiphyseal region volume (EPV), which was calculated from the distal end to the beginning of the bone diaphysis (Fig. 5c).

In each model of Fig. 5d, a compressive load was applied on the medial condyle, and the bone strength was calculated and then compared with that of the contralateral intact bone model. The graph of force, depending on defect size, was drawn to determine the critical defect size, which causes a considerably sharp reduction in bone strength. Moreover, One-Sample t-tests were applied to assess whether the mean difference between the bone strengths predicted for defects smaller or larger than the critical-sized defect was significantly different from the intact bone strength. After finding the critical defect size, larger-sized defects were flipped and intersected in the lateral condyle using ScanCAD software to analyze the effect of the defect location on the bone strength. In this step, the compressive load was distributed on both condyles with the medial and lateral compartments carrying 60 and 40 percent of the applied load, respectively^44^.

## Results

Accuracy and precision of the FE models were evaluated using the models included in the TG and EG, respectively. Therefore, to have models that could accurately predict the in vitro recorded fracture loads corresponding to TG, the elastic modulus, yield strength, and plastic modulus values were reduced incrementally up to 17% from their initial values, so that ${F}_{FE}$ accurately predicted the experimentally measured distal femoral strength with the mean prediction error, μ, not different from zero, via a paired t-test (μ = 375 N, *P* > 0.05). The 17% reduction in material properties applied to the EG models yielded μ = 129 N, with *P* > 0.05, implying the precision of the FE models with no significant difference between ${F}_{FE}$ and ${F}_{Exp}$. In addition, applying a paired t-test to the results of the fracture loads of all 14 specimens showed no significant difference between the bone strength predicted by FEM and those found via in vitro tests (*P* = 0.174). Based on the results of a 95% confidence interval of the difference between F_FE_ and F_Exp_, a maximum of 542 N was obtained in this work (see Supplementary Table S1 online), which is in the range of the previously reported data and can be considered as an acceptable error for clinical use^16^. The Pearson correlation analysis showed a high and positive correlation (r = 0.97, *P* < 0.001) between the 14 fracture loads predicted by the FEA and the corresponding fracture loads collected experimentally. Regression analysis also showed a strong linear relationship (see Supplementary Fig. S1 and Supplementary Table S2 online) between the FE predicted and experimentally recorded fracture loads for all 14 specimens ${(F}_{FE}=1.12{F}_{Exp}-0.570; {R}^{2}=0.95; P<0.001)$.

**Figure 6:**
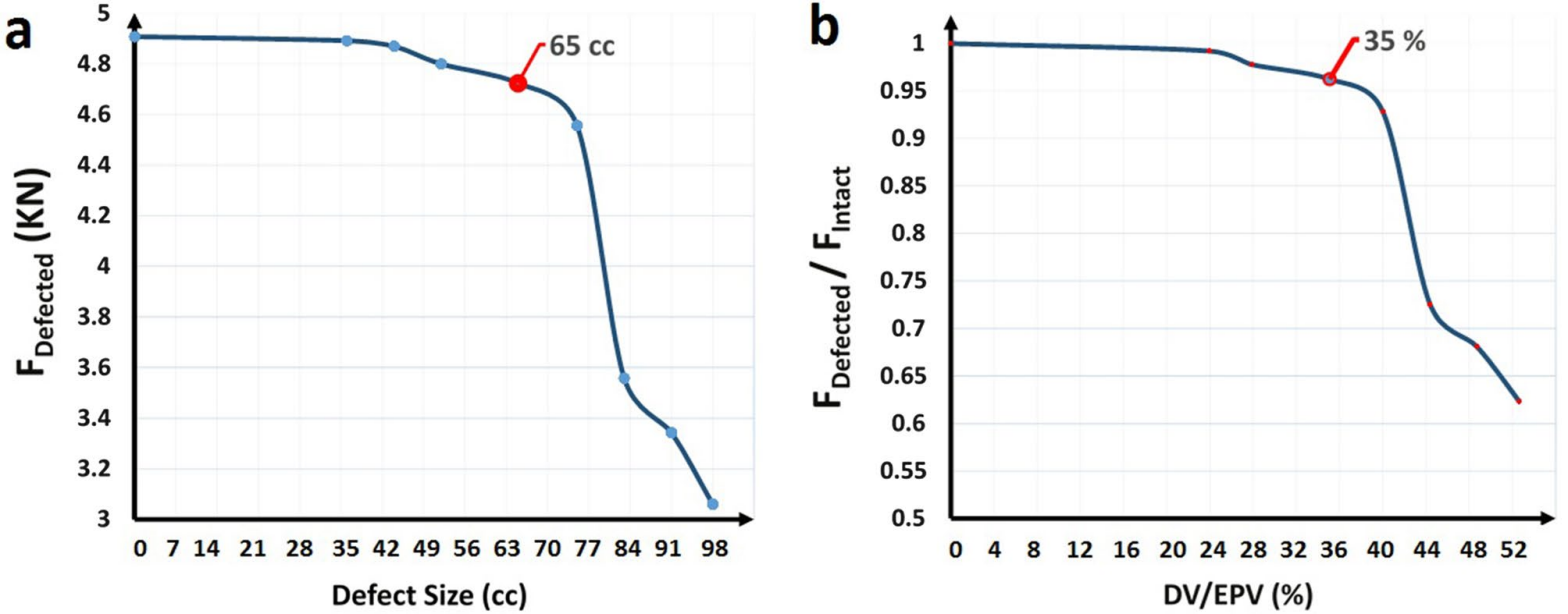
Fracture loads computed from the finite element method: (**a**) FEM fracture loads depending on defect size in the distal femur. When the defect size exceeds 65 cc, a considerable reduction occurred in the fracture load; and (**b**) FEM fracture loads of the defected bone depending on defect volume normalized to epiphyseal volume (DV/EPV). When DV/EPV exceeds 35%, there was a sharp reduction in the defected bone fracture load.

The relationship between the fracture loads predicted by FE analyses depending on defect size in the distal femur is shown in Fig. 6. As can be seen, there is a sudden jump in the graph in the vicinity of the 65 cc defect. The mean fracture loads for the four sizes of defects smaller (< 65 cc) and larger (> 65 cc) than the critical-sized defect were 4820 and 3629 N, respectively, which were compared to the fracture load of the intact bone (4908 N) with the One-Sample t-tests (see Supplementary Table S3 online). The results revealed that there is no significant difference between the mean fracture load for defect sizes until the defect is equal to 65 cc (*P* > 0.05), but the mean fracture loads of bone models with defects larger than 65 cc are significantly smaller than that of the intact bone (*P* < 0.05). By comparing FE predicted fracture loads for different defect sizes, it was found that there is not a considerable reduction, i.e., the strength reduction was not more than 5%, in the bone strength until the size of the defect volume (DV) exceeds 65 cc or 35% of the epiphyseal volume (EPV). The fracture load of the defective bone reached to 95% of the fracture load of the contralateral intact bone when the DV was 35% of EPV, but a sharp reduction in the fracture load was observed above this critical defect size value, and when the DV was 45% of EPV, the fracture load reached about 70% of that of the intact bone. Also, there was a 0.06% and 1.14% reduction in the bone strength for every 1 cc increase in the defect size for defect sizes smaller and larger than the critical-sized defect, respectively.

**Figure 7:**
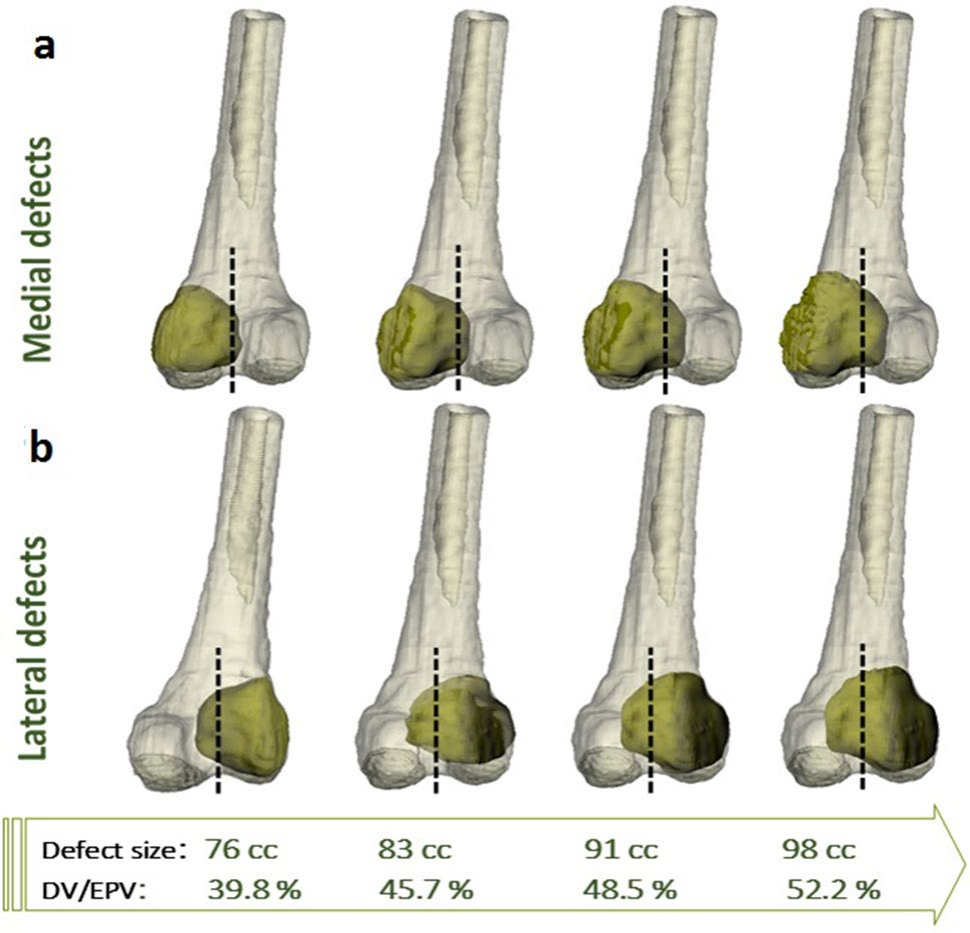
Defects larger than the critical size, i.e., 65 cc, created in (**a**) The medial condyle and (**b**) The lateral condyle. The dashed lines show the intercondylar border. The defect sizes are also given as a percent of the epiphyseal volume (EPV) occupied by the defect volume (DV).

**Figure 8:**
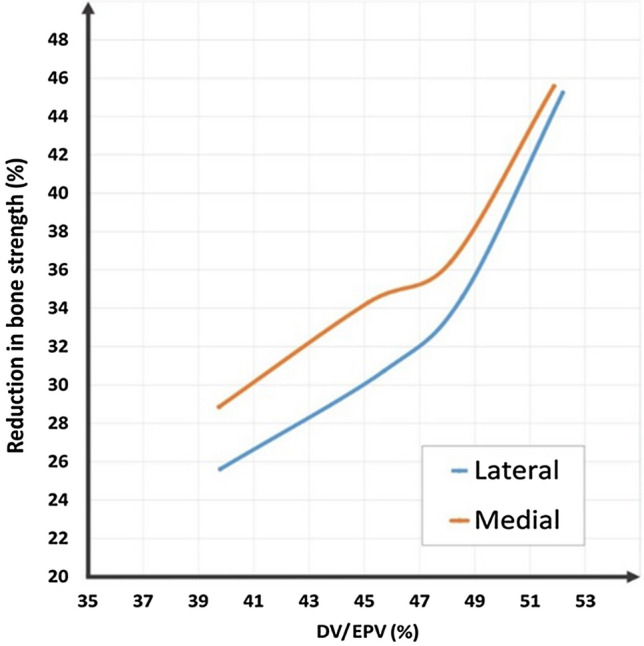
Reduction in bone strength depending on defect volume normalized to epiphyseal volume (DV/EPV) and anatomical position, which shows a greater reduction in bone strength for medially located defects with the same size and shape as those located laterally.

Defect sizes larger than the critical defect size, i.e., 76, 83, 91 and 98 cc, were modelled in both the lateral or medial compartments of an intact distal femur model (Fig. 7). In Fig. 7a,b, the black dashed lines show the border between the medial and lateral condyles. Invasion of the defect into the contralateral condyle is apparent in both medially and laterally located defects when the defect size exceeds 45% of EPV. As is shown in Fig. 8, a greater reduction in the bone strength was observed in the medially located defects compared to the laterally located ones with similar defect sizes and shapes. The same extent of reduction in bone strength was predicted for lateral defects, which were 4% on average larger than those located medially, for defect sizes in the range of 39–46% of EPV. However, when the defect size exceeded 50% of the EPV, the difference in bone strength reduction between medial and lateral defects was found to be negligible, and medial and lateral defects exhibited similar behaviours in terms of bone strength. In addition, when the defect size exceeded 47% of EPV and thus invading the contralateral condyle, a sharper reduction in bone strength was observed. Therefore, it seems that contralateral condyle involvement by a tumourous defect should be considered as another key parameter that can influence the bone fracture risk.

**Figure 9:**
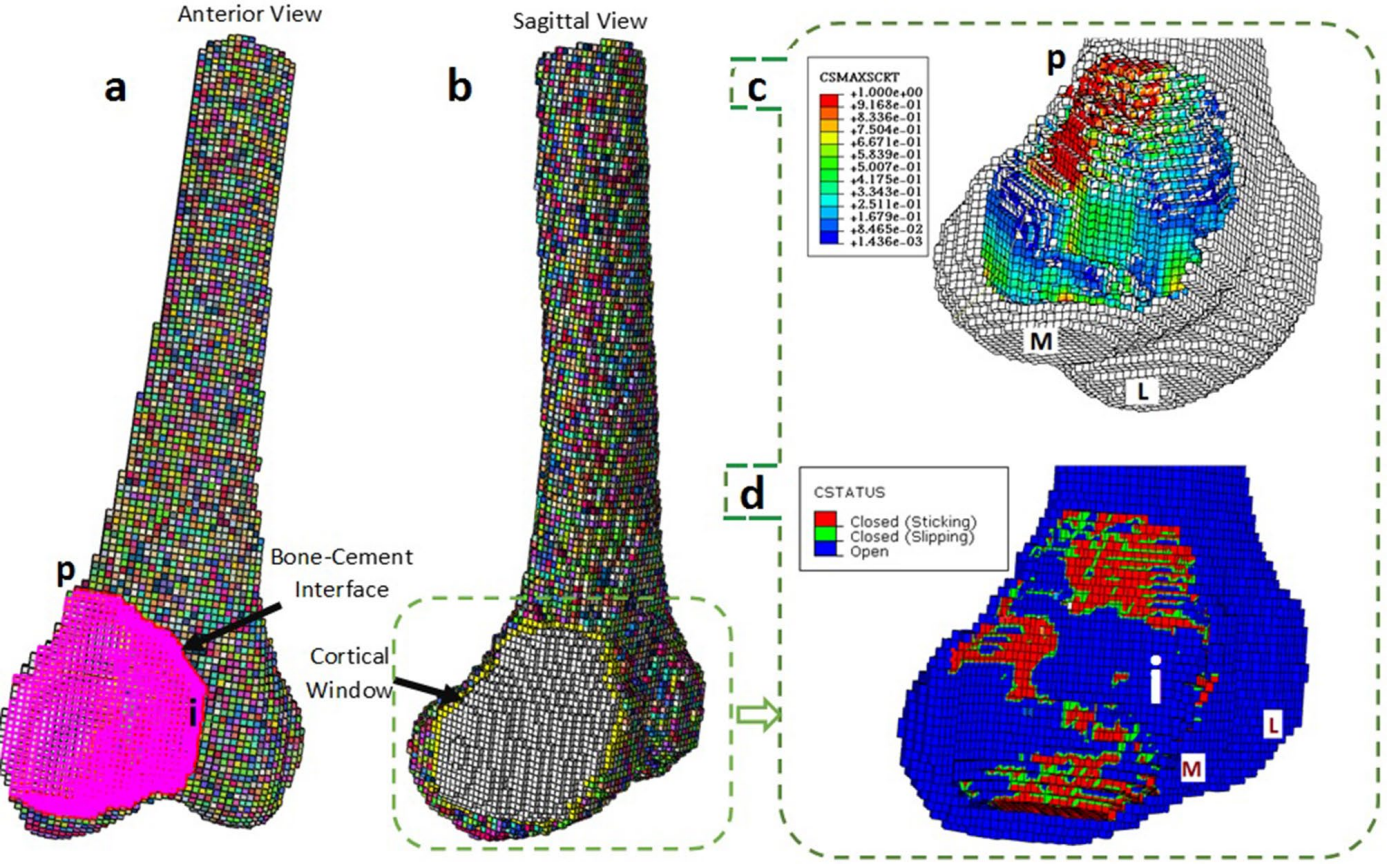
Mechanical analysis of the bone–cement interface using FEM: (**a**) Anterior view of the voxel mesh of the right distal femur, the bone–cement interface is shown in pink; (**b**) Sagittal view of the model, the cortical window border is shown in yellow; (**c**) Damage analysis indicates that the proximal end of the interface, shown by p, meets the higher values of damage initiation criterion, compared to other neighbouring regions; and (**d**) Contact analysis showed a larger debonding area of cement from bone in the most interior wall of the interface, where the cement is penetrating the contralateral condyle, indicated by i. L and M stand for lateral and medial, respectively.

Results of FEM failure analysis at the bone–cement interface can be seen in Fig. 9. Damage at the bone–cement interface was investigated by identifying regions of high stresses reaching the critical stress at damage initiation, i.e., t^0^. Therefore, as seen in Fig. 9c, which shows the contour of maximum stress criterion, the onset of damage was found to be from the proximal end of the cortical window, coloured with red, indicating that the value of $f$(see Eq. 2) is equal to one. Analysis of contact at the bone–cement interface indicated cement debonding from the interior wall of the interface, where the defect reaches the intercondylar region (Fig. 9d). In Fig. 9d, the interface is in red if the cement is still in contact with the surrounding bone, in green for the slipping contact surfaces, and in blue when the cement is debonded from the bone. As can be seen, the larger cement debonding area, indicated by ‘i’ in Fig. 9d lies in the most interior wall of the interface. The debonding area was identical for different amounts of interdigitated bone, which the results corresponding to ${q}_{int}=100\frac{\mathrm{mg}}{\mathrm{cc}}\mathrm{mm}$ can be seen in Fig. 9.

## Discussion

Management of bone defects caused by benign tumours and their associated fractures is a challenging issue in orthopaedic surgery, likely due to a lack of adequate insight of the biomechanical aspects of defected bones, as well as the absence of important biomechanical factors in their fracture risk evaluation. This study aimed to present a biomechanical approach for the assessment of bone strength following tumour curettage and to investigate the effect of defect size and location on bone strength. Moreover, the mechanics of the bone–cement interface were analyzed to shed light on the mechanisms of reconstruction failure following the procedure of choice, curettage and cement infilling, for most bone tumours, with the ultimate goal of finding the most efficient device(s) for cement augmentation.

The biomechanical approach presented here, using QCT-based FEM, showed that the accuracy and precision of the FE models are acceptable for clinical usage since no significant difference was found between fracture loads predicted by the FEM and those recorded in in vitro tests. The high accuracy of the presented FE models is likely due to considering bone non-linearity and the non-homogeneity of its material properties, both of which are significant parameters in determining bone strength^14–16,45^. Moreover, although the material properties assigned to the FE models of bone were obtained from previous research^16^, by tuning the values of the material properties, a 1:1 relation between the simulated and experimental data was found with the slope and intercept not different from one and zero, respectively, which shows the ability of the models to reliably and non-invasively predict the failure load for both intact and defective femora mimicking post-curettage conditions. The validated models were then extended to investigate the effective mechanical parameters on the bone strength, which were not been considered in previous studies^12,18,46^ or in previous fracture risk criteria^9,10^.

No specific criterion for post-operative fracture risk assessment in patients with bone tumours is currently available, and despite the important role of the tumourous defect size on fracture risk, the defect size in the presented clinical pathologic criteria is roughly measured in only one dimension from 2D X-ray images, which makes its clinical application dubious. To address this concern, in this study a biomechanical-based approach, benefiting from recent advances in imaging and modelling techniques, was introduced to find the critical defect size based on the accurate 3D volumetric size of a bone defect. The results showed that under a compressive load, there is a positive correlation between defect size and its fracture risk, which was defined by the reduction in the ultimate bone strength. Nonetheless, the correlation found was not linear, and a sharp reduction in the bone strength was predicted when the defect size exceeded 65 cc or 35% of the epiphyseal volume. This critical defect size is in good agreement with the result of the retrospective study of Hirn et al. on 146 cases having tumours around their knees, with 44 patients having tumours in their distal femur^22^. They reported that the risk of post-operative fracture was significantly greater in patients with a bony cyst larger than 60 cm^3^, based on the measurement of defect depth, width, and height, using X-ray images and with the assumption of a cylindrical or spherical shape for the tumourous defect^22^.

**Figure 10:**
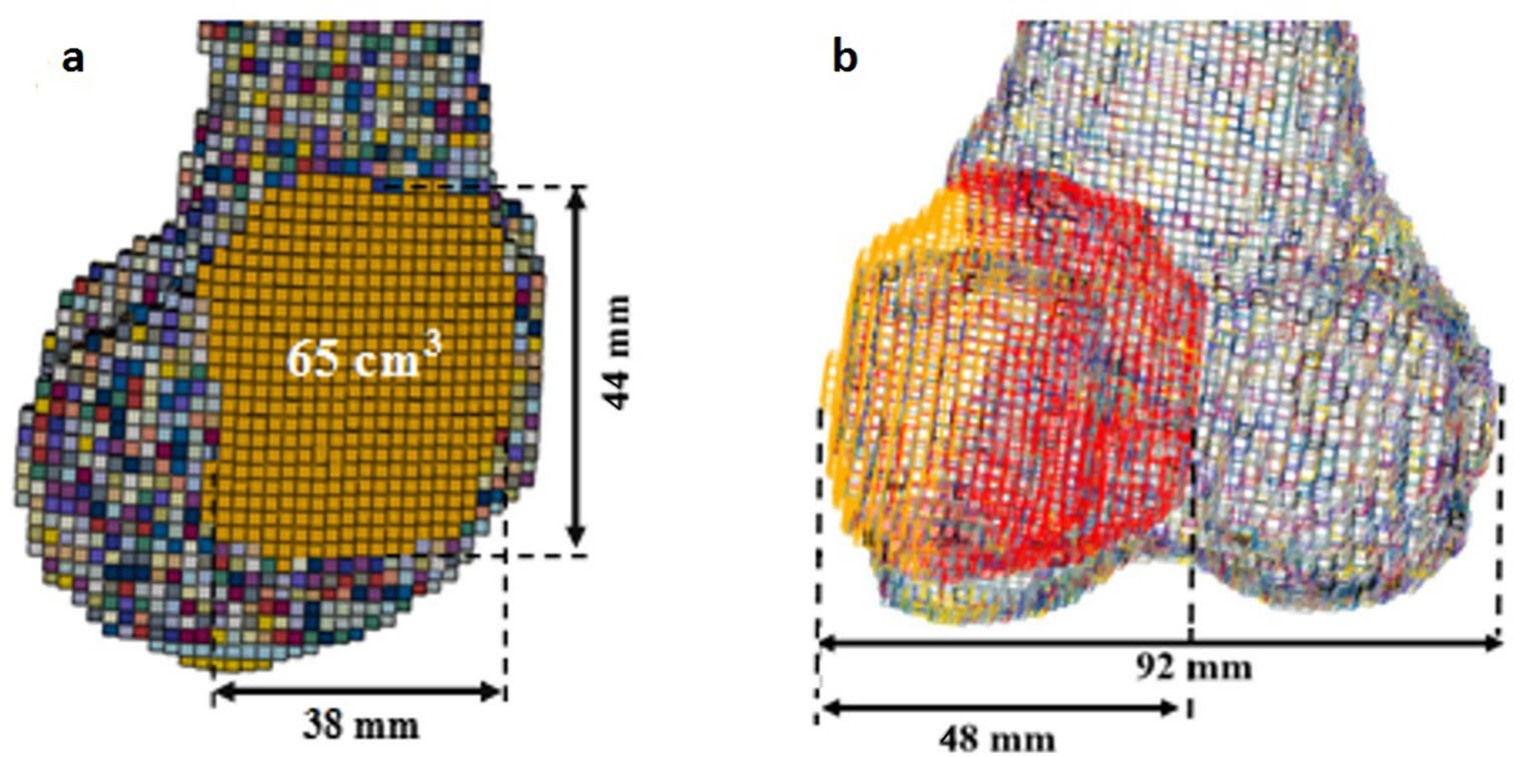
Finite element model of bone with a simulated defect of the critical size of 65 cm^3^ in (**a**) Sagittal view showing long and short diameters of 44 and 38 mm for the cortical window, respectively; and (**b**) Frontal view showing the width of the defect (48 mm) and the bone width (92 mm) making the percent of bone destruction equal to $\frac{48}{92}\times 100=52\%$.

In addition to the volumetric size, geometrical parameters of the critical defect size, such as the amount of bone cortical or bone width destruction, are also in agreement with results found for high fracture risk tumourous defects in long bones from previous biomechanical studies^5,12^. As shown in Fig. 10a, the critical defect size found in the present study, i.e., 65 cm^3^, can be assumed to have an oval shape cortical window, with a short and long diameter of 38 and 44 mm. Lin et al.^5^ reported a defect with a diameter of 30 mm or larger as critical-sized defects that need cavity infilling and fixation to reduce the risk of bone fracture when the cortical defects were simulated by circular shapes in FE models of the proximal tibia. In this study, as seen in the frontal view in Fig. 10b, the bone width is 92 mm and the defect is assumed to be a circle with a diameter of 48 mm, this means there has been bone destruction of 52% due to the critical-sized defect. Amanatullah et al. investigated the loss in the torsional integrity of synthetic distal femora related to the size of tumourous defects created by the intersection of cylinders with different radii, which made semi-circular shaped defects in the frontal view. Their results showed that a defect size destroying more than 50% of the cortical width is a critical defect, which can result in high risk of bone fracture^12^.

Anatomical location of a tumourous bone defect was thought to be another effective parameter of fracture risk, which was not considered in the currently used fracture risk criteria^9,10^. Lin et al. investigated the effect of the location of a tumourous lesion in the proximal tibia using a linear FE method^5^. They concluded that defect location is an important factor affecting fracture risk since the anteromedial wall resists fracture risk better than the other parts of the wall^5^. Results obtained from the FEA of this study also indicated the importance of defect location as an influential factor on long bone fracture risk. It was found that laterally placed defects are safer than those located in the medial part of the distal femur, assuming the same defect size and shape, while in none of the presented fracture risk criteria^9,10^ was this difference highlighted.

The most appropriate device for cement augmentation following curettage and cementation, with the aim of preventing post-operative fracture, remains a matter of debate in orthopaedics, likely due to the lack of a clear understanding of the causes of reconstruction failure and fracture^6^. To tackle this challenge, damage at the bone–cement interface was investigated using FEA in this study. The critical and vulnerable reconstruction failure positions found at the proximal end of the cortical window, and the most interior wall of the interface were in agreement with the observation of Murray et al. who tested a simulated tumourous defect reconstructed with bone cement under compression on a distal femoral specimen and took an X-ray image of the specimen after the test, which revealed a cement debonding zone at the most interior wall of the bone–cement interface^47^. Pins, screws, and plates are the most commonly used devices for cement augmentation in GCT surgery^6^. Based on the determined critical sites at the interface of bone–cement, implants that provide greater support to the most vulnerable sites for fracture should be employed. Accordingly, plates might be the best choice for cement augmentation, since a plate bridges the proximal end of the cortical window and its screws cross the interior wall of the interface, the location of the most vulnerable site of cement debonding. Other researchers employing in vitro tools^23,25,26^ have also suggested that bone plates are the most suitable implant to avoid or delay fracture in the defective areas of a long bone compared to other implants, such as pins. While some researchers have reported the benefits of cement augmentation with Steinmann pins^48,49^, other studies report no biomechanical advantages of using pins^28,47^. This work is in agreement with the latter group, that reported no advantage for cement augmentation with intramedullary pins^28,47^, since intramedullary pins neither reinforce the proximal end of the cortical window, nor the interior wall of the bone–cement, two of the most vulnerable sites of fracture initiation.

Several limitations are associated with this experimental–computational approach to finding the safest method of reconstruction following tumour curettage. First, the bone was considered as an isotropic material, despite it being anisotropic^50^. Deriving anisotropic properties of bone directly from CT scan images was impossible, and even if it were possible, for instance by using high-resolution peripheral QCT^51^, the lack of a validated failure theory for multiaxial stress–strain states of bone that could consider differences in tension and compression is a major obstacle^33^. There are limited failure theories available for isotropic modelling of bone tissue. Kayak et al.^42^ examined nine stress- and strain-based failure theories to predict the femoral fracture load using FEA with isotropic material properties. Their results showed that distortion energy and maximum shear stress failure theories were the most robust of those examined. Even complex and strain-based failure theories, such as Hoffman or Coulomb–Mohr’s theories, that consider differences between tensile and compressive failure properties do not yield better performances than distortion energy or shear stress theories^42^. However, the Drucker–Prager yield criterion^55^, which is an approximation to the Mohr–Coulomb law and a modification of the Von Mises yield criterion, is applied in some studies modelling bone as a brittle material with isotropic material properties ^52,53^. The distortion energy failure theory applied in our study has also been widely used since its validation as a robust theory for bone failure when using elastic–plastic and isotropic material properties for bone^16,33,43,54^. Warden et al.^43^ calculated femoral strength considering heterogeneous and non-linear post-yield material properties under a compressive load that was applied by an incremental displacement on the femoral head. They computed the element stress and strain, using individual element’s stress–strain relationship, similar to the relations used in this study, in conjunction with the von Mises yield criterion^43^. Most recently, a study measured hip load capacity using elastic–plastic and isotropic modeling of bone in conjunction with the distortion energy yield criterion^54^.

Since the density-material properties' relationships used in this study were originally derived for the longitudinal direction of bone^16^, in order to partially account for the effect of bone anisotropy and the error caused by ignoring it, the values of elastic modulus (E), yield stress (S), and plastic modulus (E_P_), which depend on the direction of the applied load^42^, were reduced using the tuning process, which ultimately resulted in no significant difference and error between fracture loads predicted by FEM and the in vitro experiments. However, it should be noted that there are other sources of systematic errors in the models presented in this work, ignoring anisotropy is one of them. Hence, the tuning process was employed to compensate for all possible sources of errors, e.g., error in modelling boundary conditions or the effect of the mesh size. Nonetheless, it should be noted that even though the tuning process is correcting systematic errors, due to its phenomenological approach, it remains unclear exactly what the tuning process takes into account and how much of the tuning can be attributed to a particular source of a systematic error. Another limitation of this work, caused by ignoring bone anisotropic properties, was to disregard the effects of different loading conditions on bone strength and fracture risk, which was due to the lack of accurate values of torsional and bending properties within a bone. Even though the axial force is considered as the primary factor for evaluating the risk of bone fracture^5^, torsional and bending moments are also common causes of bone fracture in the presence of a tumourous bone defect in the distal femur^12^. Hence, it seems reasonable to investigate the effects of different loading conditions in future studies to get a deeper understanding of fracture mechanisms. The second simplification that should be addressed in future studies was the lack of a standard defect shape to investigate the effect of defect size and location on the bone strength. However, it should be noted that assuming a cylindrical or spherical shape for the bone defects, knowing that it is neither, as in some previous studies^5,12^, also has its side effects. The third limitation of this work was related to analyzing the effect of defect size on the reduction in bone strength and finding the critical size of defects when located in the medial side. Even though the critical size of defects found here was in a good agreement with the clinically reported data^22^, further investigations are needed to determine if the critical size of laterally located defects is different than those located medially. The fourth limitation of this work was to investigate the usefulness of different fixation devices solely based on the results of analysis at the bone–cement interface. In order to be able to make the correct decision on the most appropriate device for cement augmentation to reduce the risk of post-operative fracture, it is better to include the fixation devices in the FE models, because many other factors, such as load transfer, the contact status between the implant and bone or cement can affect the damage initiation, stress distribution, and ultimately reconstruction failure.

## Conclusion

This study, which simultaneously employed experimental and computational approaches to study post-operative fracture risk in patients with bone-tumours, confirmed the ability of QCT-based FEM to predict the strength of both intact and defective bones, as well as to identify critical sites of reconstruction failure following tumour surgery in the distal femur. The results of this study showed that there is a considerable reduction in bone strength when a defect exceeds 35% of EPV, and also laterally defects are at lower risk of fracture compared with those located medially*.* These results should encourage researchers to think of the next steps, for instance, to look for an analytical-based, comprehensive criterion for identifying bone-tumour patients who are at high risk of post-operative fracture. Such a criterion could include detailed geometrical properties of the tumour, such as the percentage of the epiphyseal volume occupied by a tumour, its size, shape, and position, e.g., depending on if it is a medially versus laterally located defect or if the contralateral condyle is invaded by a tumoural defect, along with patients’ specific factors, such as their bone quality, daily activities, and weight. Moreover, the outcomes of damage analysis at the BCI, as a critical site for post-operative fracture initiation, highlight the need for a more analytical look at the BCI, in order to make better-informed decisions regarding the appropriate and most efficient implant for cement augmentation, all with the hope of reducing the risk of post-operative fracture in patients with bone tumours.

## Supplementary information

Supplementary Information.
