# Supplementary material for: Post-operative fracture risk assessment following tumor curettage in the distal femur: a hybrid in vitro and in silico biomechanical approach
Source: Sci Rep. 2020 Dec 7;10:21319. doi: 10.1038/s41598-020-78188-3 (PMC7721712; doi:10.1038/s41598-020-78188-3)
Supplement: Supplementary file 1 — Supplementary Information. [file 41598_2020_78188_MOESM1_ESM.pdf]

Title: Post-Operative Fracture Risk Assessment following tumor curettage in the Distal Femur: A Hybrid In vitro and In silico Biomechanical Approach

Author names and affiliations:

Azadeh Ghouchani[a], Gholamreza Rouhi[a, *], Mohammad Hosein Ebrahimzadeh[b]

[a] Faculty of Biomedical Engineering, Amirkabir University of Technology, No. 350, Hafez Ave, Valiasr Square, Tehran, Iran 1591634311

[b] Orthopaedic Research Center, Department of Orthopaedic Surgery, Mashhad University of Medical Sciences, Ahmad Abad Street, Ghaem Hospital, Mashhad, Iran

[*] **Corresponding author:** Gholamreza Rouhi

Phone: +98-2164542380

Fax: +98-2166419728

Email. grouhi@aut.ac.ir

Email addresses of the authors:

1. Azadeh Ghouchani: aghouchani@aut.ac.ir
2. Gholamreza Rouhi: grouhi@aut.ac.ir
3. Mohammad Hosein Ebrahimzadeh: ebrahimzadehmh@mums.ac.ir

Supplementary Information

The results of statistical analyses including the linear regression analysis, the paired t-test, and One-Sample t-tests are given in details in Supplementary Fig. S1 and Supplementary Tables S1-S3.

The relationship between F_{FE} and F_{Exp} for all 14 pairs using paired t-test can be seen in Supplementary Table S1. As is shown in this table, there is no significant difference between the fracture loads calculated by FEA and experiments with CI: -0.109-0.542 kN. The results also show a high and significant correlation between F_{FE} and F_{Exp} ($r=0.979$, $p<0.05$).

Supplementary Table S1 Relationships between F_{FE} and F_{Exp} using a paired t-test

	N	**Paired Differences**					**Sig. (2-tailed)**
		**Mean (N)**	**Std. Deviation (N)**	**Std. Error Mean (N)**	**95% Confidence Interval of the Difference (N)**		
					Lower	Upper	
F_{FE} - F_{Exp}	14	217	564.5	150.9	-109.0	542.9	0.174
F_{FE} &F_{Exp}	14	**Correlation (r)**			**Sig.**		
		0.979			0.001		

In Supplementary Fig. S1, the relationship between the fracture loads calculated using FEA versus those collected experimentally is shown. The details of linear regression analysis including the confidence intervals for the slope and intercept of the linear equation as well as the p-values are shown in Supplementary Table S2.

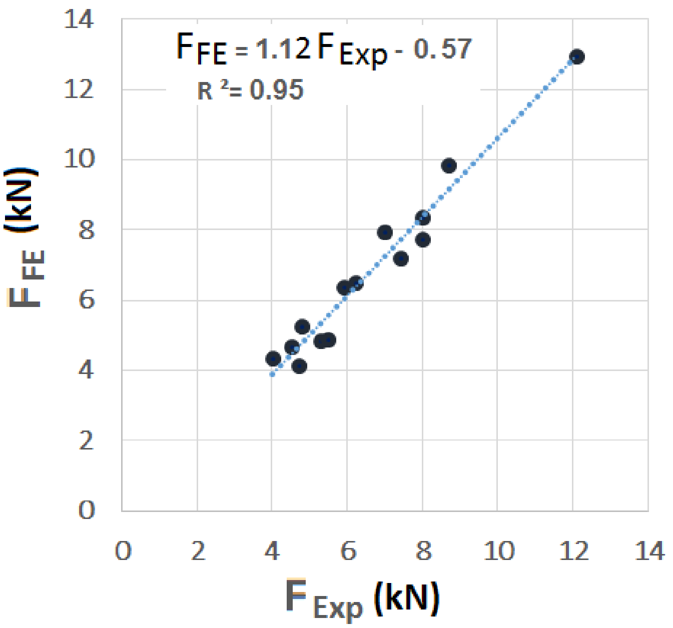

Supplementary Figure S1 Regression line showing a strong relationship between F_{FE} and F_{Exp}

Supplementary Table S2 Relationships between F_{FE} and F_{Exp} using linear regression analysis

**Pair**	N	**Linear regression equation**	**p**	**R^2**	**Sig.**	**95% Confidence Interval (CI)**	
					**for the slope of equation**		
					0.001	Lower	Upper
						0.974	1.26
					**for the intercept of equation (kN)**		
F_{FE} &F_{Exp}	14	$F_{FE} = 1.12\ F_{Exp} – 0.570$	<0.001	0.95	0.240	Lower	Upper
						-1.575	0.435

Supplementary Table S3 Results of One-Sample t-tests for the fracture loads of defects smaller (<65 cc) and larger (>65 cc) than the critical-sized defect compared with fracture load of the intact bone as the test value (4.980 kN).

	Test Value = 4.908 kN							
**Groups**	**N**	**t**	**Mean (kN)**	**Df**	**Sig. (2-tailed)**	**Mean Difference (N)**	**95% Confidence Interval of the Difference (N)**	
							Lower	Upper
< 65 cc	4	-2.337	4.820	3	.101	-88.00	-207.8252	31.8252
> 65 cc	4	-3.931	3.629	3	.029	-1278.75	-2314.0347	-243.4653
